# Associations of organisational climate and physical activity with attitudes toward sport and burnout among school administrators

**DOI:** 10.3389/fpsyg.2026.1908942

**Published:** 2026-10-20

**Authors:** Şeyda Çelik Karahançer, Yalın Kılıç, Mehmet Yaşar Kılıç

**Affiliations:** Department of Educational Sciences, Faculty of Educational Sciences, Cyprus Science University, Kyrenia, Cyprus

**Keywords:** attitudes toward sport, burnout, organisational climate, physical activity, school administrators

## Abstract

**Introduction:**

School administrators operate under substantial stress associated with heavy workloads, increasing managerial responsibilities, and organisational pressures. These conditions may be associated with higher levels of burnout. This study examined the associations among school administrators’ perceptions of organisational climate, physical activity levels, attitudes toward sport, and occupational burnout, with particular attention to their public-health relevance.

**Methods:**

The study included 223 school administrators from different school types. Data were collected using the Organisational Climate Scale, International Physical Activity Questionnaire-Short Form (IPAQ-SF), Attitudes Toward Sport Scale, and Burnout Scale. Associations among the variables were analysed using structural equation modelling (SEM). Physical activity was operationalised as the continuous total IPAQ-SF MET-min/week score derived from walking, moderate-intensity activity, and vigorous-intensity activity.

**Results:**

Positive and statistically significant associations were observed between organisational climate and attitudes toward sport and between physical activity and attitudes toward sport. Organisational climate, physical activity, and attitudes toward sport were each significantly and negatively associated with occupational burnout. In the structural model, organisational climate and physical activity accounted for 34% of the variance in attitudes toward sport, whereas organisational climate, physical activity, and attitudes toward sport jointly accounted for 32% of the variance in burnout. Bootstrap decomposition further indicated negative statistical indirect associations with burnout through attitudes toward sport for both organisational climate (βind = −0.130, bias-corrected 95% CI [−0.236, −0.081]) and physical activity (βind = −0.171, 95% CI [−0.301, −0.113]).

**Discussion:**

More favourable organisational climate, higher physical activity, and more favourable attitudes toward sport were jointly associated with lower burnout among school administrators. From a public-health perspective, these findings identify organisational and individual factors that warrant further investigation in relation to workplace mental wellbeing. They do not demonstrate that modifying these factors will prevent or reduce burnout; the effectiveness of any intervention targeting them should be evaluated in future longitudinal or experimental research.

## 1 Introduction

Insufficient physical activity remains a major and increasing global public-health concern. Pooled population-based evidence indicates that the global age-standardised prevalence of insufficient physical activity among adults increased from 23.4% in 2000 to 31.3% in 2022 (Strain et al., 2024). Sedentary behaviour and prolonged sitting have also been associated with adverse health outcomes (Park et al., 2020). Studies of university students and office workers further indicate that daily activity patterns are shaped by desk-based routines and other lifestyle or workplace factors (Carballo-Fazanes et al., 2020; Genin et al., 2018).

Another major concern in contemporary working life is burnout (Alsalhe et al., 2021). Freudenberger (1974) described staff burnout as a state of exhaustion associated with excessive occupational demands, whereas Maslach and Jackson (1981) conceptualised burnout in terms of emotional exhaustion, depersonalisation, and reduced personal accomplishment. Burnout has also been associated with depression and anxiety across occupational samples (Golonka et al., 2019; Koutsimani et al., 2019).

Regular physical activity is relevant to both physical and psychological health. It has been associated with cardiometabolic and cardiovascular benefits (Colberg et al., 2016; Ozemek et al., 2018). An umbrella review of systematic reviews of randomised controlled trials found that physical-activity interventions were associated with reductions in symptoms of depression, anxiety, and psychological distress in adults (Singh et al., 2023). Other research has linked physical activity with self-esteem, cognitive functioning, and broader wellbeing (Kim and Ahn, 2021; Mandolesi et al., 2018). These findings support the public-health relevance of physical activity without implying that the present cross-sectional study demonstrates intervention effects.

Attitudes toward sport have been described as correlates of participation in physical activity (Araújo and Dosil, 2015). Organisational climate and burnout have also been associated across occupational samples (Seyyedmoharrami et al., 2019; Lan et al., 2020), while workplace conditions and interventions can shape opportunities and support for employee physical activity (Grimani et al., 2019). However, studies integrating organisational climate, physical activity, attitudes toward sport, and burnout within a single model of school administrators remain limited. This leaves a theoretical and empirical gap at the intersection of occupational health and educational administration (Seyyedmoharrami et al., 2019; Lan et al., 2020; Grimani et al., 2019).

School administrators face distinctive occupational demands, and burnout has been examined specifically in this population. A recent systematic review of 99 empirical studies of principal burnout found that the literature has focused predominantly on antecedents and correlates, while stronger causal designs remain limited (Rogers et al., 2025). Qualitative research with school principals has likewise identified demands arising from interactions with parents, senior management, teachers, and students, as well as non-educational workload and financial concerns, as reported sources of burnout (Karaevli, 2024). However, studies integrating organisational climate, physical activity, attitudes toward sport, and burnout within a single model of school administrators remain scarce. The present study addresses this gap by examining their joint associations within educational administration and workplace public health.

### 1.1 Theoretical framework

Job Demands-Resources (JD-R) theory provides the primary occupational-health framework for integrating the organisational and individual components of the model. JD-R theory distinguishes job demands, which require sustained effort and are associated with strain, from job resources, which support goal attainment, reduce the costs of demands, and facilitate effective functioning; its health-impairment process was developed specifically to explain outcomes including burnout (Demerouti et al., 2001; Bakker and Demerouti, 2017). In the present study, organisational climate is not treated as a single job resource. Instead, it is interpreted as a contextual indicator of the extent to which resource-relevant features of the work environment, such as support, communication, structure, and constructive interpersonal relations, are perceived to be available (Demerouti et al., 2001; Prastiawan et al., 2020; Kanten and Er Ülker, 2013).

Physical activity is positioned differently from organisational climate in the theoretical model. Leisure-time physical activity is not a canonical job resource in JD-R theory and is therefore not labelled as one here (Demerouti et al., 2001; Bakker and Demerouti, 2017). Instead, it is treated as a non-work health behaviour that may coexist with recovery processes relevant to occupational wellbeing; recovery research identifies psychological detachment, relaxation, mastery, and control as distinct off-job experiences related to wellbeing (Sonnentag and Fritz, 2007). Systematic reviews have reported inverse associations between physical activity and burnout, although heterogeneity in study designs and measurement limits causal interpretation (Naczenski et al., 2017; Mincarone et al., 2024). This positioning allows the lifestyle component to be examined alongside the occupational context without extending the JD-R taxonomy beyond what the theory specifies (Demerouti et al., 2001; Bakker and Demerouti, 2017).

JD-R theory provides a direct rationale for examining organisational conditions in relation to burnout, but it does not specify attitudes toward sport as an outcome (Demerouti et al., 2001; Bakker and Demerouti, 2017). The proposed association between organisational climate and attitudes toward sport is therefore informed by complementary organisational health-behaviour research. Sonnentag and Pundt (2016) reported that organisational values, practices, and communication concerning health behaviour form a distinguishable organisational health-behaviour climate related to exercise identity and health behaviour. A systematic review of workplace physical-activity interventions similarly identified motivation and support, monitoring and feedback, information and education, and opportunities for activity as recurring organisational components of such programmes (Ramezani et al., 2022).

Importantly, the organisational climate measure used in the present study is broad rather than physical-activity-specific; consequently, the proposed organisational climate-attitude toward sport pathway is treated as a plausible contextual association rather than as a direct behavioural mechanism (Sonnentag and Pundt, 2016; Ramezani et al., 2022). Competing explanations are also plausible. Socioeconomic position is associated with domain-specific patterns of physical activity (Beenackers et al., 2012), and physical activity shows longitudinal tracking across the life course (Telama, 2009). Personality traits such as extraversion and conscientiousness are associated with physical activity (Rhodes and Smith, 2006), while positive affect has been prospectively associated with physical-activity trajectories (Qureshi et al., 2024). These factors provide credible third-variable explanations for associations involving activity and sport-related orientations and were not all measured in the present study (Beenackers et al., 2012; Telama, 2009; Rhodes and Smith, 2006).

Against this theoretical background, the present study examines contemporaneous associations among perceived organisational climate, physical activity, attitudes toward sport, and burnout in school administrators using structural equation modelling. The model tests whether organisational climate and physical activity are associated with attitudes toward sport and burnout and whether attitudes toward sport are associated with burnout. Because the data are cross-sectional, the directional paths represent theory-informed statistical specifications rather than demonstrated temporal or causal effects.

### 1.2 Hypothesis development

For H1, organisational health-behaviour research provides a more proximal rationale than JD-R theory alone. Workplace values, practices, and communication around health behaviour have been associated with exercise identity and health behaviour (Sonnentag and Pundt, 2016), and workplace physical-activity reviews identify organisational support and activity opportunities as relevant components of intervention contexts (Ramezani et al., 2022). Because the present study assesses general organisational climate rather than a sport-specific climate, a positive organisational climate-attitude toward sport association is hypothesised without assuming a direct causal mechanism (Sonnentag and Pundt, 2016; Ramezani et al., 2022).

*H1*: School administrators’ perceptions of organisational climate are positively associated with their attitudes toward sport.

Physical activity has been described as an important correlate of sport-related attitudes and behaviour (Araújo and Dosil, 2015). Participation provides direct exercise and sport experiences, and systematic-review evidence indicates that affective responses during exercise are related to later motivational constructs and future physical-activity behaviour (Rhodes and Kates, 2015).

Regular physical activity has been associated with stronger self-efficacy and physical self-perceptions (Awick et al., 2017; Mousavi Gilani and Dashipour, 2017), while self-efficacy and perceived enjoyment have been identified as predictors of subsequent physical-activity behaviour (Lewis et al., 2016). Together, these findings provide a behavioural basis for expecting more active administrators to report more favourable attitudes toward sport, although they do not establish the direction of association in the present cross-sectional data (Rhodes and Kates, 2015; Lewis et al., 2016).

Taken together, the evidence supports testing a positive association between physical activity and attitudes toward sport, while leaving temporal ordering unresolved (Araújo and Dosil, 2015; Rhodes and Kates, 2015; Lewis et al., 2016).

*H2*: School administrators’ physical activity levels are positively associated with their attitudes toward sport.

Within JD-R theory, burnout is expected to be more likely when sustained job demands are accompanied by insufficient job resources, whereas resource-rich working conditions can support functioning and reduce strain (Demerouti et al., 2001; Bakker and Demerouti, 2017). A favourable organisational climate can be interpreted as a contextual signal that resource-relevant features such as support, communication, role structure, and fair or constructive work practices are more available, without assuming that climate itself is a single job resource (Demerouti et al., 2001; Prastiawan et al., 2020; Kanten and Er Ülker, 2013).

Consistent with this interpretation, empirical studies have reported associations between organisational climate and burnout across occupational settings (Seyyedmoharrami et al., 2019; Lan et al., 2020; Giorgi et al., 2016). These findings justify testing the association among school administrators while leaving open reverse causality and the possibility that unmeasured job demands or resources contribute to both climate perceptions and burnout (Seyyedmoharrami et al., 2019; Demerouti et al., 2001; Giorgi et al., 2016).

Accordingly, more favourable perceptions of organisational climate are expected to be associated with lower occupational burnout, whereas less favourable climate perceptions are expected to be associated with higher burnout (Seyyedmoharrami et al., 2019; Lan et al., 2020; Giorgi et al., 2016).

*H3*: School administrators’ perceptions of organisational climate are negatively associated with their occupational burnout levels.

Physical activity is not modelled as a JD-R job resource; instead, it is examined as a separate health behaviour that may accompany recovery-related processes outside work (Demerouti et al., 2001; Bakker and Demerouti, 2017; Sonnentag and Fritz, 2007). Systematic-review evidence has generally reported inverse associations between physical activity and burnout, while also highlighting substantial heterogeneity and the predominance of observational designs (Naczenski et al., 2017; Mincarone et al., 2024).

Because recovery processes, social support, affective responses, and biological mechanisms were not measured in the present study, no specific mechanism is inferred from the observed physical activity–burnout association.

On this basis, physical activity is hypothesised to be negatively associated with burnout as a health-related behavioural correlate, without assuming that physical activity causally protects against or prevents burnout (Sonnentag and Fritz, 2007; Naczenski et al., 2017).

*H4*: School administrators’ physical activity levels are negatively associated with their occupational burnout levels.

Direct evidence linking attitudes toward sport with occupational burnout remains limited. Available studies in collegiate athletes and physicians have reported associations between sport- or recreation-related attitudes and burnout-related outcomes (Marangoni et al., 2023; Yıldırım and Kiraz, 2022), while sport-related orientations have been examined in relation to coping and mental-toughness constructs (Kılınç and Gürer, 2019; Özdenk and Tatlısu, 2023). Because these studies involve different populations and constructs, they provide only contextual support for H5; coping and resilience were not measured as mechanisms in the present study.

More favourable attitudes toward physical activity and sport have been reported among physically active individuals, while prior physical and sport education has been linked with continued physical activity in adulthood (Araújo and Dosil, 2015; Bendíková and Dobay, 2017). Because physical activity has itself been associated with burnout in prior literature, behavioural differences remain one possible pathway linking sport attitudes with burnout; however, that pathway was not measured in the present model (Naczenski et al., 2017).

The proposed association between attitudes toward sport and burnout can be considered in light of literature linking sport-related orientations with coping and psychological resources (Kılınç and Gürer, 2019; Özdenk and Tatlısu, 2023). Such resources may covary with emotional exhaustion, perceived efficacy, and stress regulation, but these pathways were not directly tested in the present cross-sectional model (Kılınç and Gürer, 2019; Özdenk and Tatlısu, 2023).

Taken together, the limited available literature provides a basis for testing whether more favourable attitudes toward sport are associated with lower occupational burnout, but it does not establish a generalisable or causal relationship (Marangoni et al., 2023; Yıldırım and Kiraz, 2022). Coping and resilience remain candidate mechanisms for future longitudinal or experimental research rather than mechanisms demonstrated here (Kılınç and Gürer, 2019; Özdenk and Tatlısu, 2023).

*H5*: School administrators’ attitudes toward sport are negatively associated with their occupational burnout levels.

## 2 Methods

A quantitative research approach was used to examine the associations among physical activity, perceived organisational climate, attitudes toward sport, and occupational burnout and to describe the relational structure among these variables. A relational survey design was adopted. The research model was specified in accordance with the research questions and hypotheses to represent the proposed associations among the study variables.

The model specifies direct associations of organisational climate and physical activity with attitudes toward sport and burnout, together with a negative association between attitudes toward sport and burnout.

The conceptual model was therefore designed to test how individual and organisational factors are statistically associated with burnout among school administrators (Figure 1).

**Figure 1:**
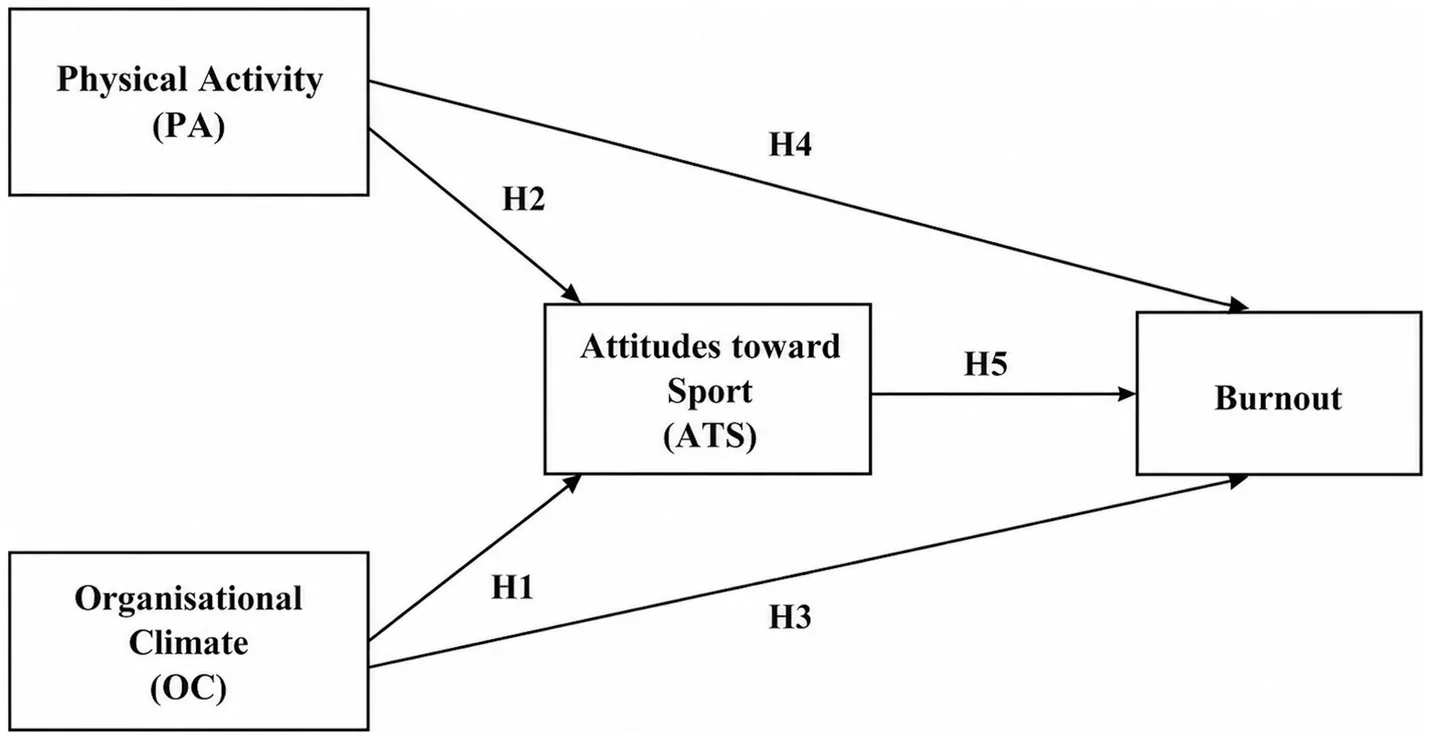
Research model.

### 2.1 Population and sample

The study population was defined using 2024 data from the Ministry of National Education of the Turkish Republic of Northern Cyprus. Excluding private educational institutions, the public-school system comprised 191 institutions: 132 primary schools, 41 secondary schools, and 18 vocational and technical schools. The corresponding eligible administrator population consisted of 483 school administrators, including 191 principals and 292 vice-principals.

School administrators working in these public educational institutions constituted the target population. Administrators from primary, secondary, and vocational–technical schools were included to ensure coverage of each school type represented in the defined population. Recruitment was conducted between 25 March and 31 May 2024 using a non-probability voluntary-response sampling approach. The electronic questionnaire invitation was disseminated through institutional communication channels, and 223 valid responses were included in the analysis. All participants were adult school administrators who provided informed consent before completing the questionnaire.

Because the number of eligible administrators who actually received or opened the invitation could not be verified, a conventional response rate could not be calculated and respondents could not be compared directly with non-respondents. Accordingly, the possibility of self-selection and non-response bias cannot be excluded. Although broad population and institutional coverage was achieved, this was not interpreted as evidence of statistical representativeness.

Several procedural measures were incorporated into the survey administration to reduce response-evaluation pressure associated with the single-source self-report design. The study purpose was explained before consent was obtained, confidentiality was assured, participation was voluntary, and no personally identifying information was collected. These procedures may reduce evaluation apprehension and socially desirable responding; however, they do not eliminate common-source variance. No temporal or source separation of the focal measures was implemented, and no dedicated marker variable was included. Common-method bias was therefore examined statistically and retained as a study limitation (Podsakoff et al., 2003).

The final sample comprised 88 principals (39.5%) and 135 vice-principals (60.5%) drawn from 170 of the 191 eligible public schools, corresponding to 89.0% institutional coverage. The represented schools included 121 primary schools, 36 secondary schools, and 13 vocational–technical schools. At the participant level, 160 administrators (71.7%) worked in primary schools, 46 (20.6%) in secondary schools, and 17 (7.6%) in vocational–technical schools. Overall, the 223 participants represented 46.2% of the known eligible administrator population of 483. This percentage is reported as population coverage rather than as a response rate.

Descriptive benchmarking was undertaken for population characteristics for which reliable reference data were available. Administrative-role distributions were identical in the population and the realised sample: principals accounted for 39.5% of both groups, while vice-principals accounted for 60.5%. At the institutional level, primary, secondary, and vocational–technical schools constituted 69.1%, 21.5%, and 9.4% of the 191 eligible schools, respectively, compared with 71.2%, 21.2%, and 7.6% of the 170 represented schools. The corresponding institutional coverage rates were 91.7% for primary schools (121/132), 87.8% for secondary schools (36/41), and 72.2% for vocational–technical schools (13/18). These comparisons indicate broad coverage and close alignment on the available population characteristics but do not establish statistical representativeness. Population-level benchmark data for administrators’ age, sex/gender, and geographical distribution were not available in the study records; therefore, direct respondent–population comparisons could not be conducted for these characteristics. Further details are provided in Supplementary Table S6.

The realised clustering structure was limited. Of the 170 represented schools, 117 contributed one administrator and 53 contributed two administrators; no school contributed more than two participants (mean cluster size = 1.31; median = 1). These cluster sizes were insufficient to support stable estimation of a multilevel SEM with school-level random effects. Potential within-school dependence was therefore examined through a complementary sensitivity analysis using school-cluster-robust sandwich standard errors for the standardised composite-score structural equations, with School_ID specified as the clustering variable.

Participant characteristics examined descriptively included age, gender, marital status, educational level, professional experience, administrative role, school type, geographical region, administrative tenure, employment status, weekly workload, and self-reported health limitations that could restrict physical activity. These characteristics are summarised in Table 1.

**Table 1:** Participant characteristics (*N* = 223).

Characteristic	Category/statistic	*n* (%) or *M* (SD)	Range
Age, years	Mean (SD)	44.61 (7.99)	28–62
Age group	44 years and under	110 (49.3)	–
	45 years and above	113 (50.7)	–
Gender	Female	105 (47.1)	–
	Male	118 (52.9)	–
Marital status	Single	57 (25.6)	–
	Married	166 (74.4)	–
Educational level	Bachelor’s	98 (43.9)	–
	Graduate	125 (56.1)	–
Professional experience, years	Mean (SD)	20.15 (8.63)	1–38
Professional experience group	1–5 years	8 (3.6)	–
	6–10 years	25 (11.2)	–
	11–15 years	39 (17.5)	–
	16 years and above	151 (67.7)	–
Administrative role	Principal	88 (39.5)	–
	Vice principal	135 (60.5)	–
School type	Primary	160 (71.7)	–
	Secondary	46 (20.6)	–
	Vocational–technical	17 (7.6)	–
Geographical region	Nicosia	81 (36.3)	–
	Kyrenia	38 (17.0)	–
	Famagusta	48 (21.5)	–
	Morphou	28 (12.6)	–
	Iskele	28 (12.6)	–
Administrative tenure, years	Mean (SD)	10.39 (5.04)	1–24
Employment status	Permanent	207 (92.8)	–
	Contract	16 (7.2)	–
Weekly workload, hours/week	Mean (SD)	43.45 (5.28)	30–60
Health limitation affecting physical activity	No	200 (89.7)	–
	Yes	23 (10.3)	–

Age groups and professional-experience categories are shown for descriptive presentation only. Continuous age and continuous years of professional experience were retained for sensitivity analyses. Health limitation denotes a self-reported limitation that could affect physical activity.

The sample included 105 women (47.1%) and 118 men (52.9%). Mean age was 44.61 years (SD = 7.99; range = 28–62; median = 45). For descriptive presentation in Table 1, age was divided at the sample median into participants aged 44 years or younger (*n* = 110, 49.3%) and those aged 45 years or older (*n* = 113, 50.7%). Continuous age was retained for the sensitivity analyses, and the dichotomised age variable was not used inferentially.

Regarding marital status, 166 participants (74.4%) were married and 57 (25.6%) were single. In terms of educational attainment, 125 participants (56.1%) had graduate-level education and 98 (43.9%) held a bachelor’s degree. Professional experience averaged 20.15 years (SD = 8.63; range = 1–38; median = 20). The grouped professional-experience categories presented in Table 1 (1–5, 6–10, 11–15, and ≥16 years) were used only for descriptive purposes, whereas continuous years of professional experience were retained in the sensitivity analyses.

Geographically, 81 participants (36.3%) worked in Nicosia, 38 (17.0%) in Kyrenia, 48 (21.5%) in Famagusta, 28 (12.6%) in Morphou, and 28 (12.6%) in Iskele. Administrative tenure averaged 10.39 years (SD = 5.04; range = 1–24), and mean weekly workload was 43.45 h (SD = 5.28; range = 30–60). Most participants held permanent appointments (*n* = 207, 92.8%), whereas 16 (7.2%) were employed on contract. Twenty-three participants (10.3%) reported a health limitation that could affect physical activity, while 200 (89.7%) reported no such limitation.

The expanded participant profile therefore provides demographic, occupational, geographical, and health-related information relevant to the interpretation of physical activity and burnout. Nevertheless, these distributions describe the realised sample and should not be interpreted as demonstrating unrestricted representativeness or generalisability.

### 2.2 Data collection instruments

Physical activity was assessed using the International Physical Activity Questionnaire–Short Form (IPAQ-SF) (Craig et al., 2003; Sağlam et al., 2010; International Physical Activity Questionnaire Research Committee, 2005). The short form records, for the preceding 7 days, the number of days and minutes per day spent in vigorous-intensity activity, moderate-intensity activity, and walking; daily sitting time is also recorded. The Turkish-language version of the IPAQ-SF, for which reliability and validity have previously been demonstrated in a Turkish sample, was used (Sağlam et al., 2010).

Responses were processed according to the revised IPAQ scoring protocol (International Physical Activity Questionnaire Research Committee, 2005). Activity bouts lasting less than 10 min/day were recoded to zero, while duration values exceeding 180 min/day for walking, moderate-intensity activity, or vigorous-intensity activity were truncated to 180 min/day. Frequency values and day/min responses were checked for valid ranges and internal consistency. In the retained dataset, activity durations ranged from 12 to 145 min/day, activity frequencies ranged from 1 to 7 days/week, and total weekly activity duration did not exceed 960 min/week. Therefore, none of the 223 retained cases required duration recoding, truncation, or exclusion under the IPAQ processing rules. The published Turkish validation was used as evidence for the language version; however, because it was not conducted specifically among school administrators in the Turkish Republic of Northern Cyprus, it was not treated as population-specific validation.

Continuous physical activity was calculated as walking MET-min/week = 3.3 × walking minutes/day × walking days/week, moderate-intensity MET-min/week = 4.0 × moderate minutes/day × moderate days/week, and vigorous-intensity MET-min/week = 8.0 × vigorous minutes/day × vigorous days/week. Component values were stored to the nearest whole MET-min/week, and total physical activity was calculated by summing the walking, moderate-intensity, and vigorous-intensity components. Sitting time was not included in the total MET-min/week score.

The resulting total MET-min/week score was analysed as a continuous, untransformed observed exogenous variable. The IPAQ low/moderate/high classification was retained only for descriptive purposes and was not used in the Pearson correlations or structural equation model. Under the 180-min/day truncation rule and a frequency range of 0–7 days/week, the processed total score has a possible range of 0–19,278 MET-min/week. Because physical activity was represented by a directly computed behavioural score rather than by a reflective multi-item construct, factor loadings, composite reliability (CR), and average variance extracted (AVE) were not calculated for this variable.

Organisational climate was assessed using the 22-item, six-dimensional Turkish form derived from the organisational-climate framework of Litwin and Stringer (1968) and used by Kanten and Er Ülker (2013). Responses were recorded on a five-point Likert scale ranging from 1 to 5. Nine negatively keyed items were reverse-scored so that higher scores consistently represented a more positive organisational climate. The prespecified dimensions were Reward (4 items), Warmth (4 items), Support/Commitment (4 items), Structure (3 items), Risk/Conflict (4 items), and Standards (3 items). Dimension and total scores were calculated as item means, with a possible range of 1–5 and an equivalent summed-score range of 22–110. Because the instrument represents a multidimensional adaptation of the broader Litwin–Stringer framework, its six-factor structure and reliability were re-evaluated in the present school-administrator sample.

Attitudes toward sport were assessed using the 25-item Attitudes toward Sport Scale developed and validated in Turkish by Şentürk (2012). The scale comprises three dimensions: Interest in Sport (12 items), Sport Lifestyle (7 items), and Active Sport Participation (6 items). All items are positively keyed and scored from 1 to 5. The possible summed score ranges from 25 to 125, with higher scores indicating more favourable attitudes toward sport. In the present study, subscale and overall mean scores ranging from 1 to 5 were used. Because the instrument was originally developed in Turkish, no translation was required. However, as the original validation sample consisted of adolescents, its three-factor structure was reassessed in the present adult school-administrator sample.

Burnout was assessed using the 22-item Maslach Burnout Inventory (Maslach and Jackson, 1981) and the Turkish adaptation reported by Ergin (1992). The Turkish form uses five response categories ranging from 0 (never) to 4 (always). The instrument comprises three dimensions: Emotional Exhaustion (9 items), Depersonalisation (5 items), and Personal Accomplishment (8 items). For the higher-order burnout model, Personal Accomplishment items were reverse-scored to represent Reduced Personal Accomplishment so that higher scores across all three dimensions consistently indicated greater burnout. Dimension means therefore ranged from 0 to 4. The established three-dimensional structure was retained as the hypothesised measurement structure and was tested empirically in the present sample.

### 2.3 Data analysis

Data were analysed using SPSS and AMOS. Preliminary screening was conducted for missing data, extreme values, response consistency, and distributional characteristics. Univariate normality was evaluated using skewness and kurtosis coefficients, all of which fell within the prespecified ±1.5 range. Structural equation modelling (SEM) was subsequently performed in AMOS using normal-theory maximum likelihood (ML) estimation applied to the sample covariance matrix.

The final SEM comprised 13 observed variables: six organisational-climate subscale mean scores, three attitudes-toward-sport subscale mean scores, three burnout subscale mean scores, and the continuous IPAQ-SF total MET-min/week score. Organisational climate, attitudes toward sport, and burnout were specified as latent constructs, whereas physical activity was entered as a single observed exogenous variable. Questionnaire items were used to calculate the prespecified subscale scores and reliability estimates but were not entered individually into the final SEM, and no item parcels were created. Because the Likert-type items were aggregated into multi-item subscale means, the resulting reflective indicators were treated as approximately continuous. For model identification, one loading for each latent construct was fixed to 1.0, while the remaining loadings and indicator residual variances were freely estimated. No correlated residuals or post-hoc paths based on modification indices were introduced.

Complete data were available for all 223 participants on the 13 variables included in the SEM. Multivariate outliers were evaluated using Mahalanobis distance. At the conservative *p* < 0.001 criterion, the critical *χ*^2^ value for 13 degrees of freedom was 34.53, whereas the largest observed Mahalanobis *D*^2^ was 27.76. Consequently, no participant was excluded as a multivariate outlier, and the final SEM sample remained *N* = 223. Multivariate normality was further examined using Mardia’s coefficients. The multivariate skewness coefficient was 9.929, *χ*^2^(455) = 369.01, *p* = 0.999, indicating no evidence of multivariate skewness. The multivariate kurtosis coefficient was 184.639, compared with the normal-theory expectation of 195, with a standardised critical ratio of −3.92 (*p* < 0.001), indicating modest multivariate platykurtosis. ML point estimates were therefore retained, while 5,000 nonparametric bootstrap resamples were used to obtain bias-corrected 95% confidence intervals for the structural coefficients.

For reproducibility, the combined measurement model comprised 12 observed subscale indicators and 27 free covariance-model parameters, yielding 51 degrees of freedom. The structural model incorporated the observed physical-activity variable and the five hypothesised structural regression paths, resulting in 31 free covariance-model parameters and 60 degrees of freedom. Structural paths are reported using the unstandardised regression coefficient (*B*), standard error (SE), standardised coefficient (*β*), bias-corrected 95% bootstrap confidence interval, AMOS *critical ratio* expressed as a Wald *z* statistic, and the corresponding two-sided *p*-value. *p*-values smaller than 0.001 are reported as *p* < 0.001 in Table 2. In this context, the term critical ratio refers to the AMOS Wald statistic and should not be confused with composite reliability.

**Table 2:** Path analysis (hypothesis testing) results.

Hypothesis	Path	*B*	SE	*β*	BC 95% CI	Wald *z*	*p*	Result
H1	OC → ATS	0.416	0.082	0.348	[0.213, 0.469]	5.08	<0.001	Supported
H2	PA → ATS	0.000713	0.000095	0.455	[0.349, 0.554]	7.54	<0.001	Supported
H3	OC → Burnout	−0.234	0.090	−0.194	[−0.338, −0.046]	−2.61	0.009	Supported
H4	PA → Burnout	−0.000265	0.000113	−0.168	[−0.308, −0.023]	−2.36	0.018	Supported
H5	ATS → Burnout	−0.378	0.083	−0.375	[−0.541, −0.201]	−4.53	<0.001	Supported

*B* = unstandardised maximum-likelihood regression coefficient; SE = normal-theory ML standard error; *β* = standardised ML SEM coefficient; BC 95% CI = bias-corrected bootstrap 95% confidence interval for *β* based on 5,000 resamples; Wald *z* = AMOS critical ratio (estimate/SE); *p* = two-sided normal-theory probability for the Wald *z* statistic. *p*-values smaller than 0.001 are reported as *p* < 0.001. OC = Organisational climate; PA = Physical activity; ATS = Attitudes toward sport. PA was operationalised as the continuous total IPAQ-SF MET-min/week score.

The structural specification also implied two indirect routes to burnout: organisational climate → attitudes toward sport → burnout and physical activity → attitudes toward sport → burnout. These indirect associations were estimated as products of the corresponding structural coefficients, with uncertainty assessed using the same 5,000-sample nonparametric bootstrap procedure. All bootstrap samples produced admissible, converged solutions, and an indirect association was considered statistically supported when its bias-corrected 95% confidence interval excluded zero. Because all variables were measured contemporaneously in a cross-sectional design, these estimates were interpreted as statistical indirect associations rather than evidence of temporal or causal mediation.

Because data collection had been completed before the analysis and the achieved sample size was therefore fixed, sample-size adequacy was evaluated through sensitivity analyses rather than retrospective observed-power calculations. The assessment was based on the complexity of the final model rather than on a hypothetical item-level model containing all 69 psychometric items. For the structural model (*N* = 223, *df* = 60), a noncentral-*χ*^2^ RMSEA sensitivity analysis at *α* = 0.05 indicated power of 0.876 to detect a change from RMSEA = 0.05 to RMSEA = 0.08; 80% power corresponded to an alternative RMSEA of approximately 0.077. Complementary noncentral-F sensitivity analyses indicated minimum detectable Cohen *f*^2^ values of 0.044 for the attitudes-toward-sport equation with two predictors and 0.050 for the burnout equation with three predictors at *α* = 0.05 and 80% power. These results indicated that the achieved sample was capable of detecting relatively small structural effects without relying on a universal cases-per-parameter rule (MacCallum et al., 1996; Wolf et al., 2013).

Before estimation of the combined measurement and structural models, scale-specific item-level confirmatory factor analyses (CFAs) were performed using the same ML estimator. Although the individual items were ordinal, each instrument used five response categories and showed no severe univariate non-normality (maximum |skewness| = 0.311; maximum |kurtosis| = 1.149). The theoretically specified correlated-factor structures were tested for organisational climate (six factors), attitudes toward sport (three factors), and burnout (three factors), and each was compared descriptively with a one-factor alternative. No correlated item residuals were specified. Internal consistency was evaluated using Cronbach’s alpha and McDonald’s omega (McDonald, 1999). Composite reliability (CR) and average variance extracted (AVE) were calculated from standardised CFA loadings, with CR ≥ 0.70 and AVE ≥ 0.50 used as reference criteria. Discriminant validity among the three higher-order reflective constructs was assessed using both the Fornell–Larcker criterion (Fornell and Larcker, 1981) and the heterotrait–monotrait ratio (HTMT) (Henseler et al., 2015), with HTMT values below 0.85 interpreted as supporting adequate discriminant validity. Item-level unstandardised loadings, standard errors, Wald *z* statistics, significance levels, and standardised loadings are reported in Supplementary Tables S1–S3.

Common-method bias was examined using several complementary sensitivity diagnostics rather than relying on a single procedure. First, all 69 psychometric items from the organisational-climate, attitudes-toward-sport, and burnout instruments were entered into an unrotated Harman-type single-factor analysis, and the variance accounted for by the first component was recorded. Second, a single-factor CFA was fitted to the 12 subscale indicators used in the combined measurement model and compared descriptively with the theoretically specified three-construct model. Third, full-collinearity variance inflation factors (VIFs) were calculated by regressing each focal composite score on the remaining focal scores. The conservative VIF criterion of 3.3 was used only as supplementary evidence because this procedure was originally developed in a PLS-SEM context (Kock, 2015). Finally, a constrained common-latent-factor sensitivity model was estimated by adding an orthogonal general method factor with an equal loading on all 12 subscale indicators. This constraint was intended to represent a uniform response tendency without allowing the method factor to absorb construct-specific covariance. None of these diagnostics was interpreted as demonstrating the absence of common-method bias (Podsakoff et al., 2003).

Pearson product–moment correlations were used to examine bivariate associations. The continuous total IPAQ-SF MET-min/week score was retained without transformation because its empirical distribution was approximately symmetric (skewness = 0.040; kurtosis = −0.268). Scores ranged from 516 to 3,296 MET-min/week, with a median of 1,782 and an interquartile range of 751.5 (Q1 = 1,402.5; Q3 = 2,154.0). Given its continuous metric and the absence of substantial univariate non-normality, Pearson correlation and ML-based SEM were considered appropriate for this variable. Table 3 reports Pearson correlations calculated from the observed/composite study scores; these coefficients should be distinguished from the latent inter-construct correlations estimated in the combined measurement model and reported for the Fornell–Larcker assessment in Table 4.

**Table 3:** Descriptive statistics, normality indicators, and observed-score pearson correlations for the study variables.

Panel A. Descriptive statistics and distributional characteristics							
Variable	*N*	Possible range	Observed range	Mean	SD	Skewness	Kurtosis
Physical activity (MET-min/week)	223	0–19,278	516–3,296	1,787.19	560.84	0.040	−0.268
Organisational climate	223	1.00–5.00	1.09–4.77	2.87	0.80	0.142	−0.728
Attitudes toward sport	223	1.00–5.00	1.00–4.92	2.95	0.89	−0.024	−0.682
Burnout	223	0.00–4.00	0.14–3.86	2.02	0.88	0.026	−0.760

Panel B. Observed-score Pearson correlations with exact two-sided *p*-values				
Variable	1. Physical activity	2. Organisational climate	3. Attitudes toward sport	4. Burnout
1. Physical activity	1.000			
2. Organisational climate	0.029 (*p* = 0.662)	1.000		
3. Attitudes toward sport	0.454 (*p* < 0.001)	0.330 (*p* < 0.001)	1.000	
4. Burnout	−0.334 (*p* < 0.001)	−0.323 (*p* < 0.001)	−0.512 (*p* < 0.001)	1.000

Panel A reports the descriptive and distributional information requested for each observed/composite study variable. Panel B reports Pearson *r* coefficients with two-sided *p*-values. The coefficients in Panel B are observed-score correlations and are therefore distinct from the latent inter-construct correlations reported in Table 4. For physical activity, the continuous total IPAQ-SF MET-min/week score was used in inferential analyses; its median was 1,782 and IQR was 751.5 (Q1 = 1,402.5; Q3 = 2,154.0).

**Table 4:** Discriminant validity of the reflective higher-order constructs.

Panel A. Fornell–Larcker criterion			
Construct	OC	ATS	BO
Organisational climate (OC)	0.711		
Attitudes toward sport (ATS)	0.360	0.848	
Burnout (BO)	−0.335	−0.522	0.824

Panel B. Heterotrait–Monotrait (HTMT) ratios			
Construct	OC	ATS	BO
Organisational climate (OC)	–		
Attitudes toward sport (ATS)	0.381	–	
Burnout (BO)	0.384	0.600	–

Panel A reports the Fornell–Larcker criterion: diagonal values are the square roots of AVE and off-diagonal values are latent inter-construct correlations from the combined measurement model. Physical activity was excluded because it was represented by a single observed IPAQ MET-min/week score rather than a reflective latent construct. Panel B reports HTMT ratios; values below 0.85 indicate adequate discriminant validity.

Overall structural-model fit was evaluated using *χ*^2^, degrees of freedom, the exact *χ*^2^ *p*-value, *χ*^2^/*df*, GFI, AGFI, CFI, TLI, NFI, RMSEA with its 90% confidence interval, and SRMR. CFI and TLI are reported as AMOS point estimates because confidence intervals for these indices are not provided in the standard AMOS ML output.

To assess the robustness of the focal structural associations to plausible demographic and occupational confounding, two supplementary covariate-adjusted sensitivity models were estimated using standardised observed composite scores with school-cluster-robust sandwich standard errors across 170 school clusters. Because age and total professional experience were strongly correlated (*r* = 0.933, *p* < 0.001), they were not entered simultaneously. The first model adjusted for continuous age, whereas the second replaced age with continuous professional experience. Both models additionally adjusted for gender, administrative role, weekly workload, school type, self-reported health limitation, administrative tenure, geographical region, and employment status. Primary school, Nicosia, female gender, principal role, permanent employment, and absence of a health limitation were used as reference categories for the corresponding indicator variables. Continuous age, professional experience, administrative tenure, and workload were retained in their original continuous metrics before standardisation. These analyses were conducted as supplementary robustness checks and did not replace the primary latent-variable SEM. Multicollinearity remained acceptable, with maximum VIF values of 2.70 in the age-adjusted model and 3.23 in the experience-adjusted model.

## 3 Results

### 3.1 Descriptive statistics

Descriptive statistics and observed-score Pearson correlations are presented in Table 3. Physical activity, organisational climate, attitudes toward sport, and burnout showed skewness and kurtosis values within the prespecified ±1.5 range. The correlations reported in Table 3 are observed-score Pearson coefficients rather than latent-variable correlations.

Mean scores were 1787.19 MET-min/week (SD = 560.84) for physical activity, 2.87 (SD = 0.80) for organisational climate, 2.95 (SD = 0.89) for attitudes toward sport, and 2.02 (SD = 0.88) for burnout. The median physical-activity score was 1,782 MET-min/week (IQR = 751.5; Q1 = 1,402.5; Q3 = 2,154.0), with an observed range of 516–3,296 MET-min/week. No transformation of the physical-activity score was applied.

Physical activity was positively associated with attitudes toward sport (*r* = 0.454, *p* < 0.001) and negatively associated with burnout (*r* = −0.334, *p* < 0.001). Organisational climate was positively associated with attitudes toward sport (*r* = 0.330, *p* < 0.001) and negatively associated with burnout (*r* = −0.323, *p* < 0.001). Attitudes toward sport were negatively associated with burnout (*r* = −0.512, *p* < 0.001). The association between physical activity and organisational climate was small and not statistically significant (*r* = 0.029, *p* = 0.662).

Multicollinearity was examined within each structural regression equation using tolerance and variance inflation factor (VIF) values (Table 5).

**Table 5:** Multicollinearity test results.

Model/predictor	Tolerance	VIF
ATS—Organisational climate	0.999	1.001
ATS—Physical activity	0.999	1.001
Burnout—Organisational climate	0.873	1.146
Burnout—Physical activity	0.778	1.285
Burnout—Attitudes toward sport	0.694	1.441

Tolerance values ranged from 0.694 to 0.999 and VIF values from 1.001 to 1.441, indicating no problematic multicollinearity among the predictors.

### 3.2 Measurement model

The multidimensional measurement structures were evaluated before the higher-order combined measurement model was estimated. For organisational climate, the theoretically specified six-correlated-factor item model fitted the data well, *χ*^2^(194) = 207.40, *p* = 0.242, *χ*^2^/*df* = 1.07, CFI = 0.995, TLI = 0.994, RMSEA = 0.018 (90% CI [0.000, 0.035]), and SRMR = 0.034. By contrast, a one-factor model showed poor fit, *χ*^2^(209) = 1040.58, *p* < 0.001, CFI = 0.692, TLI = 0.660, RMSEA = 0.134, and SRMR = 0.102. These results supported retention of the six prespecified dimensions rather than treating organisational climate as unidimensional.

Across the six organisational-climate dimensions, Cronbach’s alpha ranged from 0.823 to 0.874, McDonald’s omega from 0.829 to 0.875, CR from 0.826 to 0.875, and AVE from 0.594 to 0.649. Item-level standardised loadings ranged from 0.686 to 0.878, and every freely estimated item loading was statistically significant (*p* < 0.001). In the higher-order combined measurement model, the six dimension loadings ranged from 0.658 to 0.796; higher-order *ω* = 0.859, CR = 0.859, and AVE = 0.505. Detailed reliability and convergent-validity results are reported in Table 6, and item-level CFA estimates are reported in Supplementary Table S1.

**Table 6:** Reliability and convergent validity for the higher-order organisational climate measurement model.

Dimension	Items	Cronbach’s *α*	CR	AVE	Higher-order loading	McDonald’s *ω*
Reward	4	0.874	0.875	0.638	0.696	0.875
Warmth	4	0.852	0.854	0.597	0.699	0.854
Support/commitment	4	0.860	0.860	0.607	0.796	0.861
Structure	3	0.844	0.847	0.649	0.658	0.846
Risk/conflict	4	0.853	0.854	0.594	0.686	0.853
Standards	3	0.823	0.826	0.614	0.722	0.829
Higher-order construct	22	0.935	0.859	0.505	0.658–0.796	0.859

For attitudes toward sport, the theoretically specified three-correlated-factor item model also fitted well, *χ*^2^(272) = 305.03, *p* = 0.082, *χ*^2^/*df* = 1.12, CFI = 0.992, TLI = 0.991, RMSEA = 0.023 (90% CI [0.000, 0.036]), and SRMR = 0.033. The one-factor alternative fitted substantially less well, *χ*^2^(275) = 836.38, *p* < 0.001, CFI = 0.862, TLI = 0.850, RMSEA = 0.096, and SRMR = 0.071. Accordingly, the three-dimensional structure was retained.

For the three attitudes-toward-sport dimensions, Cronbach’s alpha ranged from 0.910 to 0.951, McDonald’s omega from 0.911 to 0.951, CR from 0.911 to 0.951, and AVE from 0.616 to 0.631. Item-level standardised loadings ranged from 0.687 to 0.882, with all freely estimated loadings significant at *p* < 0.001. In the higher-order combined measurement model, the three dimension loadings ranged from 0.728 to 0.918; higher-order *ω* = 0.881, CR = 0.884, and AVE = 0.719. The summary results are reported in Table 7, with item-level estimates in Supplementary Table S2.

**Table 7:** Reliability and convergent validity for the higher-order attitudes toward sport measurement model.

Dimension	Items	Cronbach’s *α*	CR	AVE	Higher-order loading	McDonald’s *ω*
Interest	12	0.951	0.951	0.619	0.918	0.951
Sport lifestyle	7	0.917	0.918	0.616	0.887	0.918
Active sport	6	0.910	0.911	0.631	0.728	0.911
Higher-order construct	25	0.965	0.884	0.719	0.728–0.918	0.881

For burnout, the theoretically specified three-correlated-factor item model provided good fit, *χ*^2^(206) = 243.62, *p* = 0.037, *χ*^2^/*df* = 1.18, CFI = 0.989, TLI = 0.988, RMSEA = 0.029 (90% CI [0.008, 0.042]), and SRMR = 0.033. A one-factor model fitted poorly, *χ*^2^(209) = 990.22, *p* < 0.001, CFI = 0.779, TLI = 0.756, RMSEA = 0.130, and SRMR = 0.102. The three-dimensional representation of emotional exhaustion, depersonalisation, and reduced personal accomplishment was therefore retained.

Across the burnout dimensions, Cronbach’s alpha ranged from 0.896 to 0.935, McDonald’s omega from 0.898 to 0.935, CR from 0.897 to 0.935, and AVE from 0.612 to 0.644. Item-level standardised loadings ranged from 0.655 to 0.865, and all freely estimated loadings were statistically significant (*p* < 0.001). In the higher-order combined measurement model, the dimension loadings ranged from 0.678 to 0.913; higher-order *ω* = 0.860, CR = 0.862, and AVE = 0.679. The summary results are reported in Table 8, with item-level estimates in Supplementary Table S3. The scale-specific CFAs therefore supported the prespecified multidimensional structures and did not support representing the item sets as single factors. Full comparisons between the one-factor alternatives and the theoretically specified CFA models are provided in Supplementary Table S4.

**Table 8:** Reliability and convergent validity for the higher-order burnout measurement model.

Dimension	Items	Cronbach’s *α*	CR	AVE	Higher-order loading	McDonald’s *ω*
Emotional exhaustion	9	0.933	0.934	0.612	0.913	0.935
Depersonalisation	5	0.896	0.897	0.636	0.862	0.898
Reduced Personal Accomplishment	8	0.935	0.935	0.644	0.678	0.935
Higher-order construct	22	0.957	0.862	0.679	0.678–0.913	0.860

The combined measurement model used the six organisational-climate subscale means, three attitudes-toward-sport subscale means, and three burnout subscale means as 12 observed indicators of three correlated higher-order constructs. The model contained 27 free covariance-model parameters and 51 degrees of freedom. Fit was mixed but broadly acceptable: *χ*^2^(51) = 139.79, *p* < 0.001, *χ*^2^/*df* = 2.74, GFI = 0.908, AGFI = 0.859, CFI = 0.936, TLI = 0.917, NFI = 0.903, RMSEA = 0.089 (90% CI [0.071, 0.106]), and SRMR = 0.064. The scale-specific item-level CFAs were substantially stronger than the combined higher-order model; accordingly, the latter was interpreted as an economical representation for testing relations among the multidimensional constructs rather than as evidence that the underlying item sets were unidimensional.

Discriminant validity was evaluated using two complementary criteria (Table 4). Under the Fornell–Larcker criterion, the square roots of AVE were 0.711 for organisational climate, 0.848 for attitudes toward sport, and 0.824 for burnout; each exceeded the absolute value of the corresponding latent inter-construct correlations (|*r*| = 0.335–0.522). HTMT ratios were 0.381 for organisational climate–attitudes toward sport, 0.384 for organisational climate–burnout, and 0.600 for attitudes toward sport–burnout, all below the conservative 0.85 criterion. Taken together, the Fornell–Larcker and HTMT results supported discriminant validity among the three reflective higher-order constructs. Physical activity was not included in these assessments because it was modelled as a single observed IPAQ MET-min/week behavioural index rather than a reflective latent construct.

#### 3.2.1 Common-method Bias sensitivity analyses

The Harman-type unrotated analysis of all 69 psychometric items showed that the first component accounted for 30.85% of total variance; ten components had eigenvalues greater than 1. Thus, no single component dominated the item covariance structure, although this diagnostic was interpreted cautiously because Harman’s test alone cannot exclude common-method bias. Consistent with this result, a one-factor CFA imposed on the 12 subscale indicators fitted the data poorly, *χ*^2^(54) = 729.29, *p* < 0.001, CFI = 0.512, TLI = 0.403, RMSEA = 0.237, and SRMR = 0.169, whereas the theoretically specified three-construct combined measurement model provided substantially better fit.

The auxiliary full-collinearity VIFs were 1.201 for organisational climate, 1.321 for physical activity, 1.645 for attitudes toward sport, and 1.445 for burnout, all below 3.3. In the constrained common-latent-factor sensitivity model, the equal general-method loading was estimated essentially at zero and adding this method factor did not improve model fit, Δ*χ*^2^(1) < 0.001, *p* > 0.999; the trait correlations were unchanged to three decimal places. Taken together, the diagnostics did not indicate a dominant uniform common-method component, but they cannot rule out more specific forms of common-source variance. The complete sensitivity results are reported in Supplementary Table S8.

### 3.3 Structural model

Following evaluation of the measurement models, the hypothesised structural model was tested. The latent measurement structure shown in Figure 2 was retained, while physical activity remained a single observed exogenous variable.

**Figure 2:**
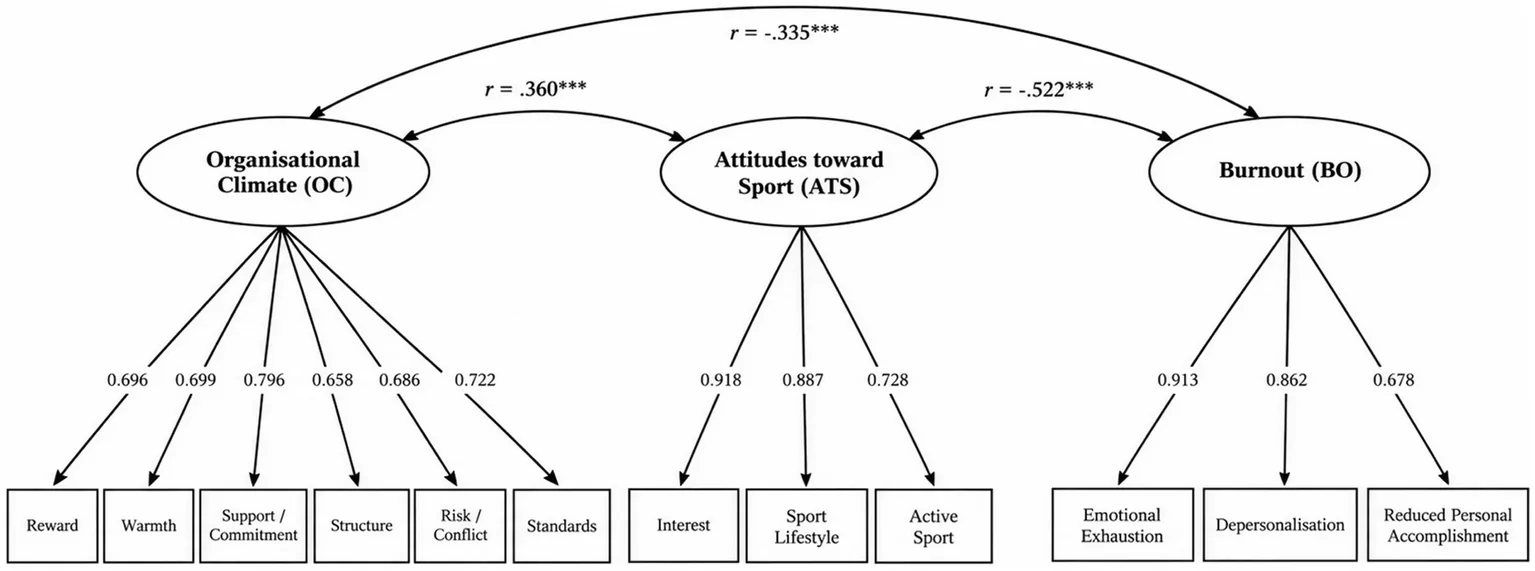
Complete measurement model. Standardised loadings are shown. All freely estimated measurement loadings were significant at *p* < 0.001. Curved double-headed arrows represent latent correlations; ^***^p < 0.001. Physical activity is a single observed IPAQ-SF MET-min/week variable and is not part of the reflective measurement model.

Figure 3 presents the structural model, and Table 2 reports the corresponding path estimates.

**Figure 3:**
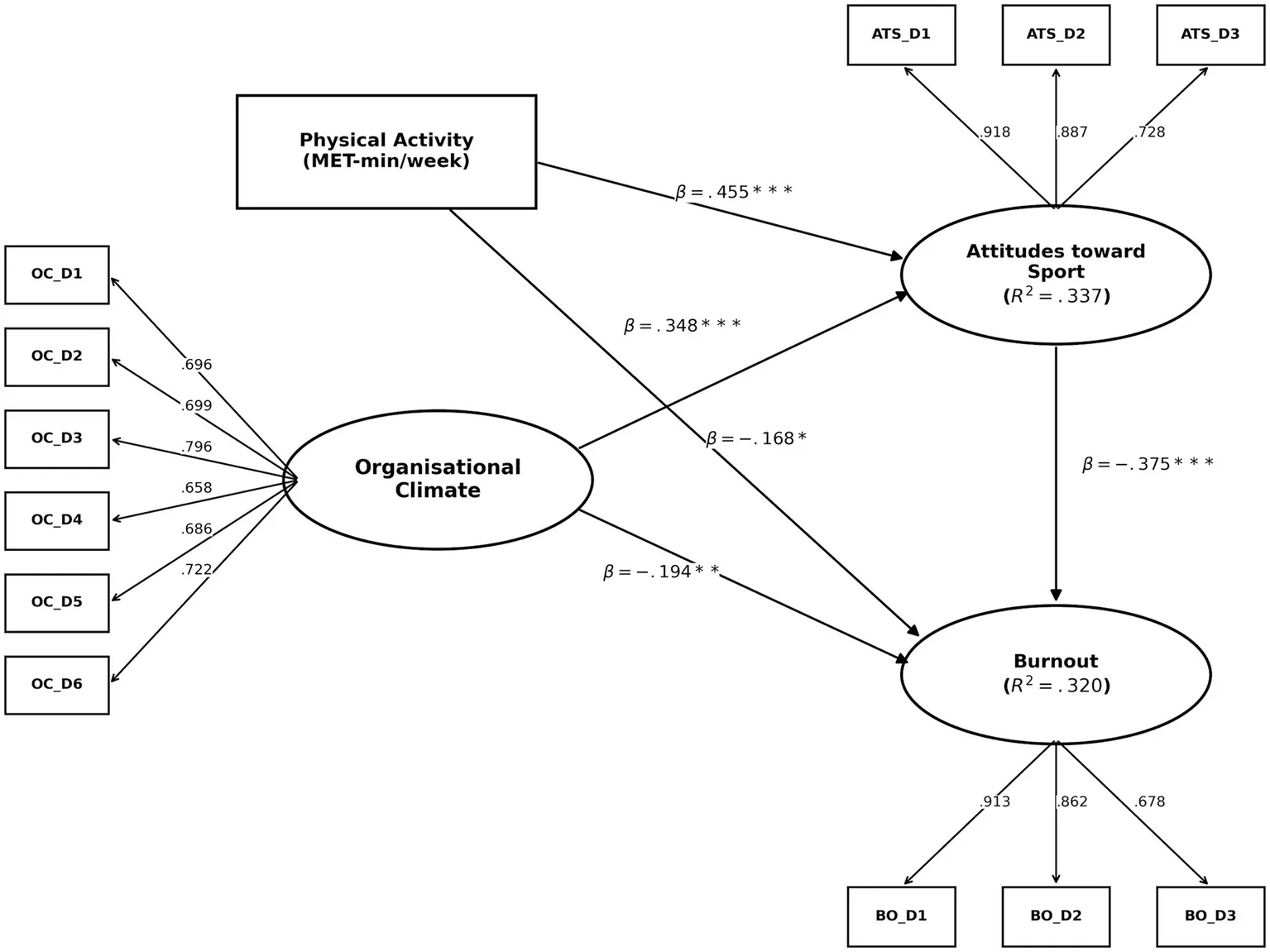
Structural model. Ellipses represent latent variables, rectangles represent observed indicators, and all coefficients shown in the figure are standardised estimates aligned with the results reported in Tables 2, 6–8. Standardised coefficients are shown. ^*^p < 0.05, ^**^p < 0.01, ^***^p < 0.001.

The structural model showed that organisational climate was positively associated with attitudes toward sport (H1: *B* = 0.416, SE = 0.082, *β* = 0.348, bias-corrected 95% CI [0.213, 0.469], *z* = 5.08, *p* < 0.001). Physical activity was also positively associated with attitudes toward sport (H2: *B* = 0.000713, SE = 0.000095, *β* = 0.455, bias-corrected 95% CI [0.349, 0.554], *z* = 7.54, *p* < 0.001).

Organisational climate was negatively associated with burnout (H3: *B* = −0.234, SE = 0.090, *β* = −0.194, bias-corrected 95% CI [−0.338, −0.046], *z* = −2.61, *p* = 0.009). Physical activity was also negatively associated with burnout (H4: *B* = −0.000265, SE = 0.000113, *β* = −0.168, bias-corrected 95% CI [−0.308, −0.023], *z* = −2.36, *p* = 0.018), as were attitudes toward sport (H5: *B* = −0.378, SE = 0.083, *β* = −0.375, bias-corrected 95% CI [−0.541, −0.201], *z* = −4.53, *p* < 0.001). Thus, all five hypothesised associations were statistically supported.

Organisational climate and physical activity accounted for 33.7% of the variance in attitudes toward sport (*R*^2^ = 0.337), while organisational climate, physical activity, and attitudes toward sport accounted for 32.0% of the variance in burnout (*R*^2^ = 0.320).

School-level clustering was examined in a supplementary sensitivity analysis. The one-way method-of-moments intraclass correlation estimate was 0.050 for physical activity, while the corresponding estimates for organisational climate, attitudes toward sport, and burnout were non-positive. When school-cluster-robust standard errors were applied across the 170 school clusters, organisational climate (*β* = 0.317, SE = 0.060, *p* < 0.001) and physical activity (*β* = 0.444, SE = 0.055, *p* < 0.001) remained positively associated with attitudes toward sport. In the burnout equation, organisational climate (*β* = −0.195, SE = 0.060, *p* = 0.001), physical activity (*β* = −0.158, SE = 0.066, *p* = 0.018), and attitudes toward sport (*β* = −0.376, SE = 0.066, *p* < 0.001) remained negatively associated with burnout. These results are reported in Supplementary Table S5.

Covariate adjustment did not alter the substantive inference (Supplementary Table S7). In the age-adjusted model, organisational climate (*β* = 0.351, cluster-robust SE = 0.062, 95% CI [0.229, 0.473], *p* < 0.001) and physical activity (*β* = 0.434, SE = 0.058, 95% CI [0.319, 0.549], *p* < 0.001) remained positively associated with attitudes toward sport (*R*^2^ = 0.349). For burnout, organisational climate (*β* = −0.215, SE = 0.061, 95% CI [−0.337, −0.094], *p* < 0.001), physical activity (*β* = −0.174, SE = 0.068, 95% CI [−0.308, −0.039], *p* = 0.012), and attitudes toward sport (*β* = −0.350, SE = 0.070, 95% CI [−0.489, −0.212], *p* < 0.001) retained their negative associations (*R*^2^ = 0.358). The experience-adjusted model produced essentially the same pattern: OC → ATS *β* = 0.347 (*p* < 0.001), PA → ATS *β* = 0.432 (*p* < 0.001), OC → burnout *β* = −0.219 (*p* < 0.001), PA → burnout *β* = −0.177 (*p* = 0.010), and ATS → burnout *β* = −0.348 (*p* < 0.001). Thus, the direction and statistical significance of all five focal associations were robust to adjustment for the measured demographic, occupational, geographical, and health-related covariates.

The structural model yielded *χ*^2^(60) = 149.70, *p* < 0.001, with *χ*^2^/*df* = 2.49. The remaining fit indices were GFI = 0.907, AGFI = 0.859, CFI = 0.938, TLI = 0.919, NFI = 0.901, RMSEA = 0.082 (90% CI [0.066, 0.099]), and SRMR = 0.056.

The fit indices showed a mixed pattern: CFI, TLI, and SRMR were within commonly used acceptable ranges, whereas RMSEA was marginally above the 0.08 benchmark. The structural model was therefore interpreted cautiously. Complete fit indices are presented in Table 9.

**Table 9:** Fit indices for the structural model.

Fit index	Good fit	Acceptable fit	Model value
*χ* ^2^	–	–	149.70
*df*	–	–	60
*p*	–	–	<0.001
*χ*^2^/*df*	0 ≤ *χ*^2^/*df* ≤ 2	2 < *χ*^2^/*df* ≤ 5	2.49
GFI	0.95 ≤ GFI ≤ 1.00	0.90 ≤ GFI < 0.95	0.907
AGFI	0.90 ≤ AGFI ≤ 1.00	0.85 ≤ AGFI < 0.90	0.859
CFI	0.95 ≤ CFI ≤ 1.00	0.90 ≤ CFI < 0.95	0.938
TLI	0.95 ≤ TLI ≤ 1.00	0.90 ≤ TLI < 0.95	0.919
NFI	0.95 ≤ NFI ≤ 1.00	0.90 ≤ NFI < 0.95	0.901
RMSEA	0 ≤ RMSEA ≤ 0.05	0.05 < RMSEA ≤ 0.08	0.082 (90% CI 0.066–0.099)
SRMR	0 ≤ SRMR ≤ 0.05	0.05 < SRMR ≤ 0.08	0.056

The structural model was estimated in AMOS by normal-theory maximum likelihood from the sample covariance matrix of 13 observed variables. The model contained 31 free covariance-model parameters and 60 degrees of freedom. Primary fit interpretation focused on *χ*^2^/*df*, CFI, TLI, RMSEA with its 90% confidence interval, and SRMR. GFI, AGFI, and NFI are retained for continuity with earlier SEM reporting but are treated as supplementary indices. Bias-corrected bootstrap confidence intervals for the structural coefficients were based on 5,000 resamples.

### 3.4 Statistical indirect associations

The two indirect associations specified in the structural model were evaluated using 5,000 nonparametric bootstrap resamples with bias-corrected 95% confidence intervals. Organisational climate showed a negative statistical indirect association with burnout through attitudes toward sport (indirect *B* = −0.157, standardised indirect *β* = −0.130, bias-corrected 95% CI [−0.236, −0.081], bootstrap *p* < 0.001). Physical activity also showed a negative statistical indirect association with burnout through attitudes toward sport (indirect *B* = −0.000270 per MET-min/week, standardised indirect *β* = −0.171, bias-corrected 95% CI [−0.301, −0.113], bootstrap *p* < 0.001). Both confidence intervals excluded zero.

The corresponding direct associations of organisational climate and physical activity with burnout remained statistically significant in the same model, indicating that the fitted model contained both direct and indirect statistical components. Because the study was cross-sectional, these indirect associations should not be interpreted as evidence of temporal or causal mediation (Table 10).

**Table 10:** Statistical indirect associations with burnout through attitudes toward sport.

Indirect association	Indirect *B*	Indirect *β*	BC 95% CI for βind	Bootstrap *p*	Inference
OC → ATS → Burnout	−0.157	−0.130	[−0.236, −0.081]	<0.001	CI excludes zero
PA → ATS → Burnout	−0.000270	−0.171	[−0.301, −0.113]	<0.001	CI excludes zero

Indirect *B* is the product of the corresponding unstandardised structural coefficients; for PA, *B* is expressed per 1 MET-min/week. Indirect *β* is the product of the corresponding standardised structural coefficients. BC 95% CI = bias-corrected 95% confidence interval from 5,000 nonparametric case-resampling bootstrap samples; all 5,000 bootstrap samples produced admissible converged solutions. Bootstrap *p*-values are two-sided. OC = Organisational climate; PA = Physical activity; ATS = Attitudes toward sport. Because the design is cross-sectional, these estimates are statistical indirect associations and should not be interpreted as temporal or causal mediation.

## 4 Discussion

The principal findings were that organisational climate and physical activity were positively associated with attitudes toward sport, while organisational climate, physical activity, and attitudes toward sport were each negatively associated with burnout. The structural model accounted for 33.7% of the variance in attitudes toward sport and 32.0% of the variance in burnout. The bootstrapped indirect associations through attitudes toward sport were also statistically supported; however, because all variables were measured contemporaneously, these estimates represent a decomposition of cross-sectional associations rather than evidence of temporal or causal mediation.

The positive association between organisational climate and attitudes toward sport is compatible with organisational health-behaviour climate research showing that workplace values, practices, and communication concerning health behaviour are related to exercise identity and health behaviour (Sonnentag and Pundt, 2016). A systematic review of workplace physical-activity interventions likewise identified motivation and support, monitoring and feedback, information and education, and opportunities for activity as recurring organisational components (Ramezani et al., 2022). Nevertheless, the organisational climate measure used in the present study was general rather than physical-activity-specific. The H1 association should therefore be interpreted as a contextual association rather than evidence that organisational climate changes sport-related attitudes. The positive association between physical activity and attitudes toward sport is also consistent with evidence that physically active individuals tend to report more favourable attitudes toward physical activity and sport (Araújo and Dosil, 2015). Because physical activity and attitudes were assessed at the same time, the present data cannot establish whether activity precedes attitudes, attitudes precede activity, or both are influenced by other factors.

The negative association between organisational climate and burnout is consistent with the Job Demands-Resources framework, in which sustained job demands and insufficient resources are linked with strain and burnout, whereas resource-relevant features of the work environment may support functioning (Demerouti et al., 2001; Bakker and Demerouti, 2017). It is also consistent with observational evidence showing associations between organisational climate and burnout-related outcomes among university employees, pharmacists, and university faculty (Seyyedmoharrami et al., 2019; Lan et al., 2020; Dinibutun et al., 2020). Principal-specific literature similarly indicates that burnout among school leaders is associated with a range of occupational and organisational demands (Rogers et al., 2025; Karaevli, 2024). These findings provide context for the observed association, but they do not establish that improving organisational climate will cause burnout to decline.

Higher physical activity was also associated with lower burnout. This finding is consistent with the systematic review by Naczenski et al. (2017), which synthesised evidence on physical activity and burnout, and with a more recent systematic review among health-care workers that generally found lower burnout risk among more physically active groups while emphasising substantial methodological heterogeneity (Mincarone et al., 2024). Toker and Biron (2012) further reported longitudinal associations among burnout, depression, and physical activity in a three-wave occupational study. These external findings support continued prospective and experimental investigation, but they should not be conflated with the present cross-sectional result. No protective, preventive, or treatment effect of physical activity can be inferred from the current data.

Attitudes toward sport were negatively associated with burnout in the fitted model. One plausible but untested explanation is that favourable sport-related attitudes may co-occur with greater engagement in physical activity: prior research has reported more favourable physical-activity and sport attitudes among physically active individuals (Araújo and Dosil, 2015), while physical activity itself has been examined in relation to burnout in longitudinal and intervention research (Naczenski et al., 2017). The present study did not test this behavioural pathway, coping processes, resilience, self-efficacy, or biological mechanisms. Accordingly, these mechanisms should be treated as hypotheses for future research rather than explanations demonstrated by the current model.

Several alternative explanations also remain plausible. Socioeconomic position is associated with domain-specific patterns of physical activity (Beenackers et al., 2012), physical activity shows longitudinal tracking across the life course (Telama, 2009), and personality traits such as extraversion and conscientiousness have been associated with physical activity (Rhodes and Smith, 2006). Positive affect has also been prospectively associated with physical-activity trajectories (Qureshi et al., 2024). These factors may contribute to the observed associations involving organisational climate, physical activity, and sport-related attitudes and were not all measured in the present study. The statistically significant indirect associations through attitudes toward sport should therefore be interpreted cautiously and not as evidence that a single psychological mechanism has been established.

The TRNC-specific interpretation has also been deliberately bounded. The present study measured administrators’ perceptions of organisational climate, physical activity, attitudes toward sport, and burnout; it did not directly measure centralised decision-making, bureaucratic burden, resource availability, or student-centred sport provision within the TRNC education system. Consequently, no explanatory claims about those contextual features are made here. The findings should be understood as estimates obtained from the realised sample of TRNC public-school administrators, and context-specific mechanisms require separate empirical investigation.

### 4.1 Limitations and future research

Several methodological limitations should be considered. First, the cross-sectional design does not establish temporal ordering or causality. Reverse causality remains plausible: administrators experiencing lower burnout may be more likely to engage in physical activity, report more favourable attitudes toward sport, or perceive their organisational climate more positively. The indirect associations should likewise be interpreted as contemporaneous statistical decompositions rather than temporal mediation.

Second, the focal variables were collected contemporaneously by self-report and are therefore vulnerable to recall error, social desirability, consistency motifs, and other forms of common-source variance. Procedural protections and the statistical sensitivity analyses did not indicate a dominant uniform method factor, but Harman’s single-factor test, full-collinearity VIFs, and the constrained common-latent-factor model are diagnostic rather than definitive. Residual common-method bias cannot be excluded.

Third, although the sample covered 46.2% of the known administrator population and 89.0% of eligible public schools, participation was voluntary and the invitation denominator could not be verified. These coverage figures therefore do not demonstrate statistical representativeness, and residual selection or non-response bias is possible. Participants were also nested within schools. Most institutions contributed only one administrator and none contributed more than two, which limited the feasibility of estimating school-level effects as a separate multilevel process; however, school-cluster-robust sensitivity analyses produced the same substantive inference as the primary SEM.

Fourth, the covariate-adjusted sensitivity analyses addressed measured demographic and occupational characteristics, but residual confounding remains possible. Socioeconomic conditions, prior sport participation and activity history, personality, positive affect, leadership style, and more detailed health characteristics may influence one or more of the observed variables (Beenackers et al., 2012; Telama, 2009; Rhodes and Smith, 2006; Qureshi et al., 2024). In addition, physical activity was represented by a computed observed IPAQ-SF total MET-min/week score rather than by a latent measurement model. Future studies should use longitudinal designs to establish temporal ordering, objective or multi-source measures where feasible, and experimental or quasi-experimental designs when intervention effectiveness is the research question.

### 4.2 Implications

The practical implications should be regarded as bounded research implications rather than intervention recommendations. Organisational climate and physical activity were associated with burnout in the present sample and therefore constitute reasonable domains for further investigation. A systematic review of workplace physical-activity interventions identified motivation and support, monitoring and feedback, information and education, and opportunities for activity as recurring intervention components (Ramezani et al., 2022); however, the present study did not evaluate any intervention. Future prospective and experimental research should determine whether changes in organisational conditions or physical activity are followed by changes in attitudes toward sport, burnout, or other indicators of occupational wellbeing before specific programmes are recommended on the basis of this model.

## 5 Conclusion

Among 223 public-school administrators in the TRNC, more favourable organisational climate and higher physical activity were associated with more favourable attitudes toward sport, while organisational climate, physical activity, and attitudes toward sport were each negatively associated with burnout. Statistically supported indirect associations through attitudes toward sport were also observed, but these estimates do not establish temporal mediation. Taken together, the findings provide an integrated account of contemporaneous organisational and health-behaviour correlates of burnout in this sample. Because the study was cross-sectional and based predominantly on self-report measures, causal, protective, and preventive conclusions are not warranted. Replication with longitudinal, multi-source, and experimental designs is required before intervention effectiveness or temporal mechanisms can be inferred.

## Data Availability

The original contributions presented in the study are included in the article/Supplementary material, further inquiries can be directed to the corresponding author/s.
