# Supplementary material for: Associations of organisational climate and physical activity with attitudes toward sport and burnout among school administrators
Source: Front Psychol. 2026 Oct 20;17:1908942. doi: 10.3389/fpsyg.2026.1908942 (PMC13727572; doi:10.3389/fpsyg.2026.1908942)
Supplement: Supplementary file 1 [file Table_1.docx]

**Supplementary Tables**

**Supplementary Table S1. Item-Level CFA Estimates for Organisational Climate**

| **Dimension** | **Item** | **B** | **SE** | **z** | **p** | **Standardised λ** |
| --- | --- | --- | --- | --- | --- | --- |
| Reward | OC1 | 1.000 | — | — | Fixed | .763 |
|  | OC2 | 1.000 | 0.088 | 11.40 | < .001 | .758 |
|  | OC3 | 0.986 | 0.079 | 12.44 | < .001 | .810 |
|  | OC4 (R) | 1.122 | 0.087 | 12.85 | < .001 | .860 |
| Warmth | OC5 | 1.000 | — | — | Fixed | .722 |
|  | OC6 | 0.923 | 0.096 | 9.63 | < .001 | .686 |
|  | OC7 | 1.102 | 0.099 | 11.13 | < .001 | .790 |
|  | OC8 (R) | 1.165 | 0.097 | 12.06 | < .001 | .878 |
| Support/Commitment | OC9 | 1.000 | — | — | Fixed | .714 |
|  | OC10 | 1.131 | 0.103 | 10.93 | < .001 | .782 |
|  | OC11 (R) | 1.054 | 0.097 | 10.81 | < .001 | .772 |
|  | OC12 (R) | 1.257 | 0.108 | 11.60 | < .001 | .843 |
| Structure | OC13 (R) | 1.000 | — | — | Fixed | .759 |
|  | OC14 (R) | 1.057 | 0.093 | 11.37 | < .001 | .805 |
|  | OC15 | 1.077 | 0.088 | 12.24 | < .001 | .849 |
| Risk/Conflict | OC16 | 1.000 | — | — | Fixed | .724 |
|  | OC17 (R) | 1.050 | 0.099 | 10.59 | < .001 | .759 |
|  | OC18 (R) | 1.029 | 0.098 | 10.47 | < .001 | .741 |
|  | OC19 (R) | 1.159 | 0.098 | 11.81 | < .001 | .852 |
| Standards | OC20 | 1.000 | — | — | Fixed | .738 |
|  | OC21 | 0.931 | 0.090 | 10.31 | < .001 | .727 |
|  | OC22 | 1.224 | 0.103 | 11.94 | < .001 | .876 |

Note. B = unstandardised loading; SE = standard error; z = Wald statistic; λ = standardised loading. The first loading for each factor was fixed at 1.00 for identification and therefore has no SE or z statistic. R denotes reverse-scored items. All freely estimated loadings were significant at p < .001.

**Supplementary Table S2. Item-Level CFA Estimates for Attitudes toward Sport**

| **Dimension** | **Item** | **B** | **SE** | **z** | **p** | **Standardised λ** |
| --- | --- | --- | --- | --- | --- | --- |
| Interest | SYT1 | 1.000 | — | — | Fixed | .687 |
|  | SYT2 | 1.118 | 0.104 | 10.73 | < .001 | .761 |
|  | SYT3 | 1.113 | 0.107 | 10.43 | < .001 | .740 |
|  | SYT4 | 1.125 | 0.105 | 10.67 | < .001 | .758 |
|  | SYT5 | 1.134 | 0.101 | 11.23 | < .001 | .797 |
|  | SYT6 | 1.128 | 0.102 | 11.03 | < .001 | .786 |
|  | SYT7 | 1.198 | 0.107 | 11.22 | < .001 | .803 |
|  | SYT8 | 1.097 | 0.101 | 10.90 | < .001 | .775 |
|  | SYT9 | 1.241 | 0.108 | 11.53 | < .001 | .823 |
|  | SYT10 | 1.235 | 0.108 | 11.48 | < .001 | .819 |
|  | SYT11 | 1.268 | 0.107 | 11.79 | < .001 | .844 |
|  | SYT12 | 1.280 | 0.109 | 11.71 | < .001 | .838 |
| Sport Lifestyle | SYT13 | 1.000 | — | — | Fixed | .740 |
|  | SYT14 | 1.038 | 0.092 | 11.33 | < .001 | .752 |
|  | SYT15 | 1.015 | 0.090 | 11.29 | < .001 | .743 |
|  | SYT16 | 1.009 | 0.088 | 11.42 | < .001 | .759 |
|  | SYT17 | 1.082 | 0.088 | 12.24 | < .001 | .808 |
|  | SYT18 | 1.121 | 0.088 | 12.68 | < .001 | .830 |
|  | SYT19 | 1.163 | 0.089 | 13.04 | < .001 | .855 |
| Active Sport | SYT20 | 1.000 | — | — | Fixed | .730 |
|  | SYT21 | 0.911 | 0.085 | 10.75 | < .001 | .726 |
|  | SYT22 | 1.007 | 0.087 | 11.55 | < .001 | .786 |
|  | SYT23 | 1.067 | 0.091 | 11.67 | < .001 | .790 |
|  | SYT24 | 1.098 | 0.088 | 12.43 | < .001 | .840 |
|  | SYT25 | 1.204 | 0.093 | 12.98 | < .001 | .882 |

Note. B = unstandardised loading; SE = standard error; z = Wald statistic; λ = standardised loading. The first loading for each factor was fixed at 1.00 for identification and therefore has no SE or z statistic. R denotes reverse-scored items. All freely estimated loadings were significant at p < .001.

**Supplementary Table S3. Item-Level CFA Estimates for Burnout**

| **Dimension** | **Item** | **B** | **SE** | **z** | **p** | **Standardised λ** |
| --- | --- | --- | --- | --- | --- | --- |
| Emotional Exhaustion | T1 | 1.000 | — | — | Fixed | .655 |
|  | T2 | 1.333 | 0.128 | 10.39 | < .001 | .796 |
|  | T3 | 1.200 | 0.123 | 9.77 | < .001 | .735 |
|  | T6 | 1.143 | 0.112 | 10.18 | < .001 | .772 |
|  | T8 | 1.181 | 0.115 | 10.23 | < .001 | .780 |
|  | T13 | 1.303 | 0.122 | 10.68 | < .001 | .818 |
|  | T14 | 1.353 | 0.126 | 10.77 | < .001 | .825 |
|  | T16 | 1.404 | 0.131 | 10.73 | < .001 | .821 |
|  | T20 | 1.377 | 0.128 | 10.75 | < .001 | .826 |
| Depersonalisation | T5 | 1.000 | — | — | Fixed | .702 |
|  | T10 | 1.244 | 0.110 | 11.35 | < .001 | .813 |
|  | T11 | 1.265 | 0.112 | 11.33 | < .001 | .814 |
|  | T15 | 1.124 | 0.102 | 10.98 | < .001 | .784 |
|  | T22 | 1.282 | 0.106 | 12.13 | < .001 | .865 |
| Reduced Personal Accomplishment | T4 (R) | 1.000 | — | — | Fixed | .795 |
|  | T7 (R) | 0.791 | 0.072 | 11.05 | < .001 | .691 |
|  | T9 (R) | 0.908 | 0.071 | 12.79 | < .001 | .777 |
|  | T12 (R) | 0.953 | 0.070 | 13.63 | < .001 | .812 |
|  | T17 (R) | 0.949 | 0.067 | 14.10 | < .001 | .828 |
|  | T18 (R) | 0.963 | 0.070 | 13.78 | < .001 | .818 |
|  | T19 (R) | 0.958 | 0.065 | 14.68 | < .001 | .853 |
|  | T21 (R) | 0.965 | 0.068 | 14.22 | < .001 | .835 |

Note. B = unstandardised loading; SE = standard error; z = Wald statistic; λ = standardised loading. The first loading for each factor was fixed at 1.00 for identification and therefore has no SE or z statistic. R denotes reverse-scored items. All freely estimated loadings were significant at p < .001.

**Supplementary Table S4. Scale-Specific CFA Model Fit and One-Factor Comparisons**

| **Scale** | **Model** | **χ²** | **df** | **p** | **χ²/df** | **CFI** | **TLI** | **RMSEA (90% CI) / SRMR** |
| --- | --- | --- | --- | --- | --- | --- | --- | --- |
| Organisational climate | One-factor | 1040.58 | 209 | < .001 | 4.98 | 0.692 | 0.660 | 0.134 [0.126, 0.142] / 0.102 |
|  | Six correlated factors | 207.40 | 194 | .242 | 1.07 | 0.995 | 0.994 | 0.018 [0.000, 0.035] / 0.034 |
| Attitudes toward sport | One-factor | 836.38 | 275 | < .001 | 3.04 | 0.862 | 0.850 | 0.096 [0.089, 0.103] / 0.071 |
|  | Three correlated factors | 305.03 | 272 | .082 | 1.12 | 0.992 | 0.991 | 0.023 [0.000, 0.036] / 0.033 |
| Burnout | One-factor | 990.22 | 209 | < .001 | 4.74 | 0.779 | 0.756 | 0.130 [0.122, 0.138] / 0.102 |
|  | Three correlated factors | 243.62 | 206 | .037 | 1.18 | 0.989 | 0.988 | 0.029 [0.008, 0.042] / 0.033 |
| Combined measurement model | Three higher-order constructs | 139.79 | 51 | < .001 | 2.74 | 0.936 | 0.917 | 0.089 [0.071, 0.106] / 0.064 |

Note. Scale-specific models were estimated from item-level responses using AMOS-style normal-theory maximum likelihood. The combined measurement model used the 12 prespecified subscale mean scores as indicators of three correlated higher-order constructs. No correlated item residuals were introduced.

**Supplementary Table S5. School-Cluster-Robust Sensitivity Analysis of the Structural Associations**

| **Path** | **Primary SEM β** | **Robust β** | **Cluster-robust SE** | **95% CI** | **p** | **Inference** |
| --- | --- | --- | --- | --- | --- | --- |
| OC → ATS | .348 | .317 | .060 | [.200, .435] | < .001 | Unchanged |
| PA → ATS | .455 | .444 | .055 | [.336, .553] | < .001 | Unchanged |
| OC → Burnout | −.194 | −.195 | .060 | [−.313, −.076] | .001 | Unchanged |
| PA → Burnout | −.168 | −.158 | .066 | [−.288, −.028] | .018 | Unchanged |
| ATS → Burnout | −.375 | −.376 | .066 | [−.506, −.246] | < .001 | Unchanged |

Note. Sensitivity models used standardised observed composite scores and school-cluster-robust sandwich standard errors with School_ID as the clustering unit (170 clusters). They were used solely to assess the influence of within-school dependence and do not replace the primary latent-variable SEM. One-way method-of-moments ICC estimates were .050 for PA and non-positive for OC, ATS, and Burnout; non-positive sample ICC estimates were interpreted as indicating no detectable positive between-school variance.

**Supplementary Table S6. Descriptive Comparison of the Realised Sample with Available Population Benchmarks**

| **Benchmark** | **Known population** | **Realised sample / represented institutions** | **Descriptive comparison** |
| --- | --- | --- | --- |
| Administrative role: Principal | 191/483 (39.5%) | 88/223 (39.5%) | Closely aligned |
| Administrative role: Vice principal | 292/483 (60.5%) | 135/223 (60.5%) | Closely aligned |
| School type: Primary (institution level) | 132/191 (69.1%) | 121/170 represented schools (71.2%) | 91.7% of eligible primary schools represented |
| School type: Secondary (institution level) | 41/191 (21.5%) | 36/170 represented schools (21.2%) | 87.8% of eligible secondary schools represented |
| School type: Vocational–technical (institution level) | 18/191 (9.4%) | 13/170 represented schools (7.6%) | 72.2% of eligible vocational–technical schools represented |
| Overall administrator coverage | 483 eligible administrators | 223 participants | 46.2% population coverage; not a response rate |
| Overall institutional coverage | 191 eligible public schools | 170 represented schools | 89.0% institutional coverage |

Note. Population figures are the 2024 Ministry-based counts used to define the study population. Role comparisons are administrator-level; school-type comparisons are institution-level because eligible administrator counts by school type were unavailable. Population benchmarks for age, sex/gender, and geographical distribution were unavailable and were not compared. Because participation was voluntary and the invitation denominator was unknown, coverage percentages and descriptive alignment should not be interpreted as a response rate or proof of statistical representativeness.

**Supplementary Table S7. Covariate-Adjusted Sensitivity Analysis of the Structural Associations**

| **Sensitivity model** | **Path** | **Adjusted β** | **Cluster-robust SE** | **95% CI** | **p** |
| --- | --- | --- | --- | --- | --- |
| Age-adjusted | OC → ATS | 0.351 | 0.062 | [0.229, 0.473] | < .001 |
|  | PA → ATS | 0.434 | 0.058 | [0.319, 0.549] | < .001 |
|  | OC → Burnout | -0.215 | 0.061 | [-0.337, -0.094] | < .001 |
|  | PA → Burnout | -0.174 | 0.068 | [-0.308, -0.039] | .012 |
|  | ATS → Burnout | -0.350 | 0.070 | [-0.489, -0.212] | < .001 |
| Experience-adjusted | OC → ATS | 0.347 | 0.062 | [0.224, 0.469] | < .001 |
|  | PA → ATS | 0.432 | 0.058 | [0.317, 0.548] | < .001 |
|  | OC → Burnout | -0.219 | 0.061 | [-0.340, -0.098] | < .001 |
|  | PA → Burnout | -0.177 | 0.068 | [-0.311, -0.043] | .010 |
|  | ATS → Burnout | -0.348 | 0.070 | [-0.486, -0.210] | < .001 |

Note. Supplementary sensitivity models used standardised observed composite scores and school-cluster-robust sandwich standard errors with School_ID as the clustering unit (170 clusters). Both models adjusted for gender, administrative role, weekly workload, school type, health limitation, administrative tenure, geographical region, and employment status. Because age and professional experience were strongly correlated (r = .933, p < .001), the age-adjusted model included continuous age but not professional experience, whereas the experience-adjusted model included continuous professional experience but not age. Reference categories were female, principal, primary school, Nicosia, permanent employment, and no health limitation. Maximum VIF values were 2.70 and 3.23, respectively. Age-adjusted R² values were .349 for attitudes toward sport and .358 for burnout; experience-adjusted R² values were .350 and .356, respectively. These analyses are robustness checks and do not replace the primary latent-variable SEM.

**Supplementary Table S8. Common-Method Bias Sensitivity Diagnostics**

| **Diagnostic** | **Statistic** | **Result** | **Interpretation** |
| --- | --- | --- | --- |
| Harman-type unrotated analysis (69 psychometric items) | Variance accounted for by first component | 30.85%; 10 components with eigenvalues > 1 | No single component dominated; not interpreted as a definitive CMB test. |
| Single-factor CFA of the 12 subscale indicators | χ²(54), CFI, TLI, RMSEA, SRMR | 729.29, .512, .403, .237, .169 | A single general factor reproduced the covariance structure poorly. |
| Full-collinearity VIF (auxiliary diagnostic) | OC / PA / ATS / Burnout | 1.201 / 1.321 / 1.645 / 1.445 | All values < 3.3; criterion used as supplementary rather than conclusive evidence. |
| Constrained orthogonal common latent factor | Equal method loading; Δχ²(1) | Loading ≈ 0; Δχ² < .001, p > .999 | No evidence of a uniform general response factor; construct correlations unchanged to 3 decimals. |
